# Evaluation of Subclinical Vascular Disease in Diabetic Kidney Disease: A Tool for Personalization of Management of a High-Risk Population

**DOI:** 10.3390/jpm12071139

**Published:** 2022-07-14

**Authors:** Christodoula Kourtidou, Vasileios Rafailidis, Garyfallia Varouktsi, Efthimios Kanakis, Vassilios Liakopoulos, Timoleon-Achilleas Vyzantiadis, Maria Stangou, Smaragdi Marinaki, Konstantinos Tziomalos

**Affiliations:** 1 First Propedeutic Department of Internal Medicine, Medical School, Aristotle University of Thessaloniki, AHEPA Hospital, 1 Stilponos Kyriakidi Street, 54636 Thessaloniki, Greece; efthimioskanakis@yahoo.gr (E.K.); ktziomalos@yahoo.com (K.T.); 2 Department of Radiology, Medical School, Aristotle University of Thessaloniki, AHEPA Hospital, 54636 Thessaloniki, Greece; billraf@hotmail.com; 3 Department of Nephrology, Medical School, Aristotle University of Thessaloniki, AHEPA Hospital, 54636 Thessaloniki, Greece; varoukts@med.uth.gr (G.V.); vliak@auth.gr (V.L.); 4 First Department of Microbiology, Medical School, Aristotle University of Thessaloniki, 54124 Thessaloniki, Greece; avyz@med.auth.gr; 5 Department of Nephrology, Medical School, Aristotle University of Thessaloniki, Hippokration Hospital, 54642 Thessaloniki, Greece; mstangou@auth.gr; 6 Department of Nephrology and Renal Transplantation, Medical School, National and Kapodistrian University of Athens, Laiko Hospital, 11527 Athens, Greece; smaragdimarinaki@yahoo.com

**Keywords:** type 2 diabetes mellitus, diabetic kidney disease, diabetic nephropathy, arterial stiffness, pulse wave velocity, carotid atherosclerosis, peripheral arterial disease, ankle-brachial index, cardiovascular disease, personalization

## Abstract

Background: Patients with diabetic kidney disease (DKD) are at increased risk for cardiovascular events but traditional risk factors do not fully explain this association. Evaluation of subclinical vascular disease might improve risk stratification and management of these patients. The aim of the study was to compare the prevalence of markers of arterial stiffness, carotid atherosclerosis and peripheral arterial disease between patients with DKD and patients with type 2 diabetes mellitus (T2DM) and preserved kidney function. Methods: We prospectively enrolled patients with DKD and age- and gender-matched patients with T2DM but without DKD (estimated glomerular filtration rate < and ≥60 mL/min/1.73 m^2^, respectively). The presence of arterial stiffness was evaluated by measuring pulse wave velocity (PWV), augmentation index (AIx), AIx adjusted to a heart rate of 75 beats/min (AIx@75) and central systolic, diastolic, pulse and mean blood pressure. The presence of carotid atherosclerosis was evaluated by measuring carotid stenosis, carotid intima-media thickness and maximal plaque thickness. The presence of PAD was evaluated with the measurement of ankle-brachial index (ABI). Results: Forty patients with T2DM were included in the study (mean age 71.6 ± 8.9 years). The prevalence of cardiovascular risk factors was similar in patients with and without DKD. PWV was higher in the former (9.8 ± 5.5 and 6.6 ± 4.4 m/s, respectively; *p* < 0.05) and carotid stenosis of the left carotid artery was also greater in patients with DKD (36.5 ± 12.6 and 22.1 ± 17.2%, respectively; *p* < 0.05). Other markers of arterial stiffness and carotid atherosclerosis and ABI did not differ between patients with DKD and those without DKD. Conclusions: Patients with DKD appear to have more pronounced arterial stiffness and carotid atherosclerosis than patients with T2DM and preserved kidney function despite the similar prevalence of traditional cardiovascular risk factors in the two groups. Therefore, evaluating the presence of subclinical vascular disease in these patients could be a useful tool for the personalization of their management.

## 1. Introduction

Diabetic kidney disease (DKD) is observed in approximately 40% of patients with type 2 diabetes mellitus (T2DM) and is the leading cause of end-stage renal disease (ESRD) in Western countries [1,2]. Patients with DKD are at increased risk for cardiovascular events, which is similar to those with a history of myocardial infarction [3,4]. In T2DM, excess mortality is largely limited to patients with DKD [4,5].

Given the substantial cardiovascular morbidity associated with DKD, identification of patients with DKD who are at the highest risk for cardiovascular events is pivotal for prevention of such events. However, traditional risk factors, including hypertension, dyslipidemia and obesity, do not fully explain the increased cardiovascular risk of patients with DKD [6,7]. Accordingly, additional markers are needed for accurate risk stratification in this population [6,8]. In this context, several studies showed that the presence of subclinical vascular disease is strongly related to the incidence of cardiovascular events in patients with T2DM [9,10,11]. However, only few studies compared the prevalence of multiple markers of subclinical vascular disease between patients with DKD and those with T2DM and preserved kidney function [12,13].

The aim of the present study was to compare the prevalence of markers of arterial stiffness, carotid atherosclerosis and peripheral arterial disease (PAD) between patients with DKD and patients with T2DM and preserved kidney function.

## 2. Patients and Methods

We performed a cross-sectional study in which we enrolled all adult patients with DKD who visited the Internal Medicine Outpatients Clinic of the First Propedeutic Department of Internal Medicine between August 2021 and April 2022. Age- and gender-matched patients with T2DM but without DKD who visited the same outpatient clinic during this period were enrolled as controls. Patients with acute kidney injury were excluded from the study.

Demographic data (age, sex), history of cardiovascular risk factors (hypertension, atrial fibrillation, smoking, alcohol intake, family history of cardiovascular disease (CVD)) and history of concomitant CVD (coronary heart disease, ischemic stroke, heart failure) were recorded. Anthropometric parameters (weight, height, waist circumference), heart rate and systolic and diastolic blood pressure (BP) were also measured.

In all patients, serum levels of total cholesterol, low-density lipoprotein cholesterol, high-density lipoprotein cholesterol, triglycerides, HbA_1c_ and creatinine were determined in fasting blood samples and the urinary albumin/creatinine ratio was determined in a morning spot urine sample. Glomerular filtration rate (GFR) was estimated with the Chronic Kidney Disease Epidemiology Collaboration equation [14]. According to the estimated GFR (eGFR), the study population was divided into: (a) patients with DKD (eGFR < 60 mL/min/1.73 m^2^) and (b) patients without DKD (eGFR ≥ 60 mL/min/1.73 m^2^).

The presence of arterial stiffness was evaluated by measuring pulse wave velocity (PWV), augmentation index (AIx), AIx adjusted to a heart rate of 75 beats/min (AIx@75), central systolic blood pressure (cSBP), central diastolic blood pressure (cDBP), central pulse pressure (cPP) and central mean pressure (cMP) using a Sphygmocor device (Atcor Medical, Sydney, Australia) [11]. Measurements were performed in the morning, in the supine position, after at least 10 min of rest and after fasting for at least 4 h [11]. Two measurements were performed, and the mean was recorded. If the two measurements differed by >0.5 m/s in PWV or by >5% in AIx, a third measurement was performed, and the median was recorded [11].

The presence of carotid atherosclerosis was evaluated by measuring carotid stenosis, carotid intima-media thickness (cIMT) and maximal plaque thickness (MPT) using a Logiq S8 ultrasound device (GE Healthcare, Milwaukee, Wisconsin). Carotid stenosis was measured according to the European Carotid Surgery Trial (ECST) method [15]. The cIMT was measured between the intimal-luminal and the medial-adventitial interfaces of the carotid artery wall [16]. The MPT was measured as the height of the largest plaque from any segment of the right and left common carotid arteries [17].

The presence of PAD was evaluated with the measurement of the ankle-brachial index (ABI) in both legs [18].

All data were analyzed using IBM SPSS Statistics for Windows (version 27: Armonk, NY, USA: IBM Corp). Data are presented as percentages for categorical variables and as mean and standard deviation for continuous variables. Differences in categorical and continuous variables between groups were assessed using the Chi-square test and the independent samples t-test, respectively. Correlations between continuous variables were evaluated using Pearson’s correlations. In all cases, a two-tailed *p* < 0.05 was considered significant.

The study was conducted according to the Declaration of Helsinki and was approved by the Ethics Committee of the Medical School of the Aristotle University of Thessaloniki. All patients provided written informed consent.

## 3. Results

Patients’ characteristics are shown in Table 1. A total of 40 patients with T2DM were included in the study. Eighteen patients had DKD (66.7% males, age 77.4 ± 6.5 years) and 22 patients did not have DKD (72.7% males, age 76.9 ± 7.9 years). The urinary albumin/creatinine ratio was higher in patients with DKD than in patients without DKD (280.6 ± 473.9 and 41.4 ± 50.3 mg/g, respectively; *p* < 0.05). In contrast, demographic, anthropometric and laboratory characteristics did not differ between the two groups. The prevalence of cardiovascular risk factors, including hypertension, smoking, established CVD and lipid and HbA_1c_ levels, were also similar in patients with and without DKD.

Markers of subclinical vascular disease in patients with DKD and in patients without DKD are shown in Table 2. PWV was higher in the former (9.8 ± 5.5 and 6.6 ± 4.4 m/s, respectively; *p* < 0.05). Other markers of arterial stiffness (AIx, AIx@75, cSBP, cDBP, cPP and cMP) did not differ between patients with DKD and patients without DKD. Carotid stenosis of the left carotid artery was greater in the former (36.5 ± 12.6 and 22.1 ± 17.2%, respectively; *p* < 0.05). Other markers of carotid atherosclerosis did not differ between patients with DKD and patients without DKD. The ABI in both legs was similar in the two groups (Table 2).

In the total study population, the eGFR correlated with carotid stenosis of the left carotid artery (r = −0.397, *p* < 0.05) and did not correlate with other markers of subclinical vascular disease.

## 4. Discussion

In the present study, PWV was higher in patients with DKD than in patients with T2DM and preserved kidney function. Given that risk factors for arterial stiffness, particularly age and BP, did not differ between the two groups, it appears that DKD is independently associated with arterial stiffness. Even though other markers of arterial stiffness did not differ between patients with and without DKD, it is widely accepted that PWV is the gold standard for the evaluation of this marker of target organ damage [11]. Similar to our findings, a previous study reported that diabetic patients with ESRD have higher PWV than patients with T2DM but without DKD [19]. Our study expands these results in patients with less advanced DKD. In contrast, another study in T2DM patients reported no difference in PWV between patients with eGFR ≥ and <60 mL/min/1.73 m^2^ [12]. However, in the latter study, eGFR was estimated using the Modification of Diet in Renal Disease (MDRD) Study equation [12]. In contrast, we used the CKD-EPI equation to measure eGFR since the latter equation has less bias, improved precision and greater accuracy than the MDRD Study equation, especially in patients with GFR ≥ 60 mL/min/1.73 m^2^ [20,21]. Notably, PWV not only predicts cardiovascular events but two prospective studies reported that it is also independently associated with the development and progression of albuminuria in patients with T2DM [22,23]. It has been suggested that increased arterial stiffness results in higher systolic pressure and greater pressure fluctuations in renal arteries, leading to renal microvascular damage and renal dysfunction [24]. More studies are needed to evaluate whether the measurement of PWV in patients with T2DM has prognostic implications for either cardiovascular events or the progression of DKD. On the other hand, it should be noted that measurement of PWV is time-consuming and needs better standardization and that our study possibly lacked power to firmly establish an association between arterial stiffness and DKD. Moreover, there was a trend for higher prevalence of hypertension in patients with DKD, which is a major risk factor for arterial stiffness and might have contributed to the higher PWV in these patients.

In our study, patients with DKD had more advanced carotid atherosclerosis than patients without DKD even though cardiovascular risk factors did not differ between the two groups. The pathogenesis of atherosclerosis in patients with DKD is multifactorial, with both conventional and emerging risk factors playing a role [7]. Recently, sterol-O-acyltransferase-1, an enzyme that esterifies free cholesterol, the NLRP3 inflammasome pathway and the interleukin-33/ST2 pathway have also been implicated in the atherogenetic process in these patients [25,26,27,28,29,30,31]. Several previous reports observed greater atherosclerotic burden in the carotid arteries of patients with DKD than in diabetic patients with preserved renal function [32,33,34,35]. However, most of these studies were performed in non-Caucasians and none evaluated multiple indices of carotid atherosclerosis (i.e., cIMT, degree of stenosis and plaque thickness) [32,33,34,35]. Interestingly, prospective studies suggest that carotid atherosclerosis predicts, not only cardiovascular events, but also renal function decline in patients with DKD [36,37]. Therefore, evaluation of carotid atherosclerosis in this population might be a useful tool for optimizing the management of risk factors to prevent cardiorenal morbidity, which might potentially include novel agents [38]. However, in our study, stenosis in only the left carotid artery differed between patients with and without DKD, whereas other markers of carotid atherosclerosis were similar in the two groups. In addition, small degrees of stenosis might be due to error. Therefore, it is important to validate these findings in larger, independent cohorts.

In the present study, the ABI did not differ between patients with and without DKD. ABI is the recommended test for diagnosing PAD and is an independent predictor of cardiovascular events [10,14]. To the best of our knowledge, this is the first study that evaluated the association between DKD and ABI in Caucasian patients with T2DM. A study in Japanese patients showed no correlation between ABI and eGFR [39], whereas a report from Taiwan revealed an independent association between ABI and DKD [40]. Given these limited data, particularly in non-Asians, and that ABI is an inexpensive, non-invasive, easily performed and sensitive marker of cardiovascular risk [10,14], more studies are needed to characterize the usefulness of ABI as a risk stratification tool in patients with DKD.

Even though patients with DKD are considered to be already at very high cardiovascular risk and guidelines recommend aggressive medical treatment with high-dose, high-intensity statins and aspirin [41], the identification of subclinical vascular disease in this population might have implications for their management. Indeed, several approaches have shown benefit in very high-risk populations, including double antiplatelet treatment [42], combined of antiplatelet agents and low-dose direct-acting anticoagulants [43], anti-inflammatory agents [44] or more aggressive lipid-lowering treatment with the addition of ezetimibe and PCSK-9 inhibitors to achieve LDL < 40 mg/dL [45,46,47]. In addition, even though guidelines recommend aggressive medical treatment in all patients with diabetes mellitus, many patients do not adhere to treatment and many physicians do not adhere to guidelines [48]. It is possible that the identification of subclinical arterial disease might improve adherence of both patients and physicians.

In conclusion, patients with DKD appear to have more pronounced arterial stiffness and carotid atherosclerosis than patients with T2DM and preserved kidney function despite the similar prevalence of traditional cardiovascular risk factors in the two groups. Therefore, the presence of DKD might represent an independent risk factor for vascular disease and the evaluation of the presence of subclinical vascular disease in these patients could be a useful tool for the personalization of their treatment and the prevention of cardiovascular events. However, the present study included relatively few patients and larger studies are needed to confirm our findings.

## Figures and Tables

**Table 1:** Characteristics of patients with diabetic nephropathy and of patients without diabetic nephropathy (data shown are percentages or mean ± SD).

	Patients with Diabetic Nephropathy (*n* = 18)	Patients without Diabetic Nephropathy (*n* = 22)	*p*
Age (years)	77.4 ± 6.5	76.9 ± 7.9	0.873
Males (%)	66.7	72.7	0.945
Systolic blood pressure (mmHg)	139.8 ± 17.3	141.9 ± 12.0	0.650
Diastolic blood pressure (mmHg)	81.6 ± 13.9	81.9 ± 12.8	0.936
Heart rate	71.3 ± 9.1	74.7 ± 10.9	0.293
Hypertension (%)	100.0	77.3	0.093
Type 2 diabetes mellitus duration (years)	15.1 ± 8.8	11.4 ± 6.6	0.133
Smoking (current/past, %)	5.6/44.4	22.7/36.4	0.318
Package-years	36.2 ± 19.2	52.5 ± 35.2	0.223
Alcohol intake (units/week)	0.50 ± 1.3	0.91 ± 1.6	0.398
Atrial fibrillation (%)	38.9	13.6	0.142
Family history of cardiovascular disease (%)	27.8	18.2	0.732
Coronary heart disease (%)	44.4	36.4	0.846
Ischemic stroke (%)	22.3	4.5	0.220
Heart failure (%)	27.8	9.1	0.259
Weight (kg)	81.2 ± 19.4	84.7 ± 13.9	0.515
Body mass index (kg/m^2^)	28.4 ± 6.1	29.4 ± 3.9	0.558
Waist circumference (cm)	104.8 ± 13.8	106.4 ± 9.9	0.680
Total cholesterol (mg/dL)	129.9 ± 37.3	123.1 ± 53.8	0.655
Low-density lipoprotein cholesterol (mg/dL)	59.7 ± 23.8	57.5 ± 50.7	0.868
High-density lipoprotein cholesterol (mg/dL)	45.1 ± 20.4	39.5 ± 12.4	0.295
Triglycerides (mg/dL)	153.8 ± 83.4	135.1 ± 51.8	0.390
HbA_1c_ (%)	6.9 ± 1.2	7.5 ± 1.3	0.221
Estimated glomerular filtration rate (mL/min/1.73 m^2^)	36.8 ± 11.3	87.0 ± 11.9	<0.001
Urinary albumin/creatinine ratio (mg/g)	280.6 ± 473.9	41.4 ± 50.3	0.024
Treatment with statins (%)	100.0	100.0	(-)
Treatment with antiplatelet agents (%)	55.6	36.4	0.371
Treatment with antihypertensive agents (%)	100.0	77.3	0.093

**Table 2:** Markers of subclinical vascular disease in patients with diabetic nephropathy and in patients without diabetic nephropathy (data shown are mean ± SD).

	Patients with Diabetic Nephropathy (*n* = 18)	Patients without Diabetic Nephropathy (*n* = 22)	*p*
Ankle-brachial index (left)	1.07 ± 0.23	1.09 ± 0.22	0.806
Ankle-brachial index (right)	1.08 ± 0.21	1.06 ± 0.27	0.791
Pulse wave velocity (m/sec)	9.8 ± 5.5	6.6 ± 4.4	0.039
Augmentation index (%)	29.6 ± 12.3	24.5 ± 11.9	0.201
Augmentation index adjusted to a heart rate of 75 beats/min (%)	29.7 ± 11.2	27.3 ± 13.3	0.556
Central systolic blood pressure (mmHg)	127.1 ± 11.8	128.3 ± 10.2	0.741
Central diastolic blood pressure (mmHg)	76.2 ± 10.7	81.4 ± 13.5	0.198
Central mean blood pressure (mmHg)	97.2 ± 10.8	103.3 ± 11.8	0.097
Central pulse pressure (mmHg)	48.9 ± 15.4	46.9 ± 11.8	0.639
Carotid stenosis (left)(%)	36.5 ± 12.6	22.1 ± 17.2	0.024
Carotid intima-media thickness (left)	0.82 ± 0.17	0.79 ± 0.30	0.779
Maximal plaque thickness (left)	0.25 ± 0.11	0.17 ± 0.12	0.087
Carotid stenosis (right) (%)	31.3 ± 17.8	21.8 ± 18.6	0.177
Carotid intima-media thickness (right)	0.93 ± 0.26	0.89 ± 0.32	0.718
Maximal plaque thickness (right)	0.22 ± 0.11	0.25 ± 0.29	0.756

## Data Availability

Data are available upon request.

## References

[1] Reutens A.T. (2013). Epidemiology of diabetic kidney disease. Med. Clin. N. Am..

[2] Fernández Fernández B., Elewa U., Sánchez-Niño M.D., Rojas-Rivera J.E., Martin-Cleary C., Egido J., Ortiz A. (2012). 2012 update on diabetic kidney disease: The expanding spectrum, novel pathogenic insights and recent clinical trials. Minerva Med..

[3] Sarnak M.J., Levey A.S., Schoolwerth A.C., Coresh J., Culleton B., Hamm L.L., McCullough P.A., Kasiske B.L., Kelepouris E., Klag M.J. (2003). American Heart Association Councils on Kidney in Cardiovascular Disease, High Blood Pressure Research, ClinicalCardiology, and Epidemiology and Prevention. Kidney disease as a risk factor for development of cardiovascular disease: A statement from the American Heart Association Councils on Kidney in Cardiovascular Disease, High Blood Pressure Research, Clinical Cardiology, and Epidemiology and Prevention. Circulation.

[4] Afkarian M., Sachs M.C., Kestenbaum B., Hirsch I.B., Tuttle K.R., Himmelfarb J., de Boer I.H. (2013). Kidney disease and increased mortality risk in type 2 diabetes. J. Am. Soc. Nephrol..

[5] Bruno G., Merletti F., Bargero G., Novelli G., Melis D., Soddu A., Perotto M., Pagano G., Cavallo-Perin P. (2007). Estimated glomerular filtration rate, albuminuria and mortality in type 2 diabetes: The Casale Monferrato study. Diabetologia.

[6] Coll B., Betriu A., Martínez-Alonso M., Borràs M., Craver L., Amoedo M.L., Marco M.P., Sarró F., Junyent M., Valdivielso J.M. (2010). Cardiovascular risk factors underestimate atherosclerotic burden in chronic kidney disease: Usefulness of non-invasive tests in cardiovascular assessment. Nephrol. Dial. Transplant..

[7] Gansevoort R.T., Correa-Rotter R., Hemmelgarn B.R., Jafar T.H., Heerspink H.J., Mann J.F., Matsushita K., Wen C.P. (2013). Chronic kidney disease and cardiovascular risk: Epidemiology, mechanisms, and prevention. Lancet.

[8] Koji Y., Tomiyama H., Ichihashi H., Nagae T., Tanaka N., Takazawa K., Ishimaru S., Yamashina A. (2004). Comparison of ankle-brachial pressure index and pulse wave velocity as markers of the presence of coronary artery disease in subjects with a high risk of atherosclerotic cardiovascular disease. Am. J. Cardiol..

[9] Irie Y., Katakami N., Kaneto H., Nishio M., Kasami R., Sakamoto K., Umayahara Y., Sumitsuji S., Ueda Y., Kosugi K. (2013). The utility of carotid ultrasonography in identifying severe coronary artery disease in asymptomatic type 2 diabetic patients without history of coronary artery disease. Diabetes Care.

[10] Fowkes F.G., Murray G.D., Butcher I., Heald C.L., Lee R.J., Chambless L.E., Folsom A.R., Hirsch A.T., Dramaix M., Ankle Brachial Index Collaboration (2008). Ankle brachial index combined with Framingham Risk Score to predict cardiovascular events and mortality: A meta-analysis. JAMA.

[11] Townsend R.R., Wilkinson I.B., Schiffrin E.L., Avolio A.P., Chirinos J.A., Cockcroft J.R., Heffernan K.S., Lakatta E.G., McEniery C.M., Mitchell G.F. (2015). American Heart Association Council on Hypertension. Recommendations for Improving and Standardizing Vascular Research on Arterial Stiffness: A Scientific Statement From the American Heart Association. Hypertension.

[12] Sjöblom P., Nystrom F.H., Länne T., Engvall J., Östgren C.J. (2014). Microalbuminuria, but not reduced eGFR, is associated with cardiovascular subclinical organ damage in type 2 diabetes. Diabetes Metab..

[13] Choi S.W., Yun W.J., Kim H.Y., Lee Y.H., Kweon S.S., Rhee J.A., Choi J.S., Shin M.H. (2010). Association between albuminuria, carotid atherosclerosis, arterial stiffness, and peripheral arterial disease in Korean type 2 diabetic patients. Kidney Blood Press. Res..

[14] Levey A.S., Stevens L.A., Schmid C.H., Zhang Y.L., Castro A.F., Feldman H.I., Kusek J.W., Eggers P., Van Lente F., Greene T. (2009). A new equation to estimate glomerular filtration rate. Ann. Intern. Med..

[15] European Carotid Surgery Trialists’ Collaborative Group (1991). MRC European Carotid Surgery Trial: Interim results for symptomatic patients with severe (70–99%) or with mild (0–29%) carotid stenosis. Lancet.

[16] de Groot E., vanLeuven S.I., Duivenvoorden R., Meuwese M.C., Akdim F., Bots M.L., Kastelein J.J. (2008). Measurement of carotid intima-media thickness to assess progression and regression of atherosclerosis. Nat. Clin. Pract. Cardiovasc. Med..

[17] Johri A.M., Nambi V., Naqvi T.Z., Feinstein S.B., Kim E.S.H., Park M.M., Becher H., Sillesen H. (2020). Recommendations for the Assessment of Carotid Arterial Plaque by Ultrasound for the Characterization of Atherosclerosis and Evaluation of Cardiovascular Risk: From the American Society of Echocardiography. J. Am. Soc. Echocardiogr..

[18] Gerhard-Herman M.D., Gornik H.L., Barrett C., Barshes N.R., Corriere M.A., Drachman D.E., Fleisher L.A., Fowkes F.G., Hamburg N.M., Kinlay S. (2017). 2016 AHA/ACC Guideline on the Management of Patients With Lower Extremity Peripheral Artery Disease: A Report of the American College of Cardiology/American Heart Association Task Force on Clinical Practice Guidelines. Circulation.

[19] El Ghoul B., Daaboul Y., Korjian S., ElAlam A., Mansour A., Hariri E., Samad S., Salameh P., Dahdah G., Blacher J. (2017). Etiology of End-Stage Renal Disease and Arterial Stiffness among Hemodialysis Patients. BioMed. Res. Int..

[20] Stevens L.A., Schmid C.H., Greene T., Zhang Y.L., Beck G.J., Froissart M., Hamm L.L., Lewis J.B., Mauer M., Navis G.J. (2010). Comparative performance of the CKD Epidemiology Collaboration (CKD-EPI) and the Modification of Diet in Renal Disease (MDRD) Study equations for estimating GFR levels above 60 mL/min/1.73 m^2^. Am. J. Kidney Dis..

[21] Michels W.M., Grootendorst D.C., Verduijn M., Elliott E.G., Dekker F.W., Krediet R.T. (2010). Performance of the Cockcroft-Gault, MDRD, and new CKD-EPI formulas in relation to GFR, age, and body size. Clin. J. Am. Soc. Nephrol..

[22] Cardoso C.R.L., Leite N.C., Salles G.C., Ferreira M.T., Salles G.F. (2018). Aortic stiffness and ambulatory blood pressure as predictors of diabetic kidney disease: A competing risks analysis from the Rio de JaneiroType 2 Diabetes Cohort Study. Diabetologia.

[23] Zhang X., Low S., Sum C.F., Tavintharan S., Yeoh L.Y., Liu J., Li N., Ang K., Lee S.B., Tang W.E. (2017). Arterial stiffness is an independent predictor for albuminuria progression among Asians with type 2 diabetes—A prospective cohort study. J. Diabetes Complicat..

[24] O’Rourke M.F., Safar M.E. (2005). Relationship between aortic stiffening and microvascular disease in brain and kidney: Cause and logic of therapy. Hypertension.

[25] Liu X., Ducasa G.M., Mallela S.K., Kim J.J., Molina J., Mitrofanova A., Wilbon S.S., Ge M., Fontanella A., Pedigo C. (2020). Sterol-O-acyltransferase-1 has a role in kidney disease associated with diabetes and Alport syndrome. Kidney Int..

[26] Hai Q., Smith J.D. (2021). Acyl-Coenzyme A: Cholesterol Acyltransferase (ACAT) in Cholesterol Metabolism: From Its Discovery to Clinical Trials and the Genomics Era. Metabolites.

[27] Luo M., Opoku E., Traughber C.A., Hai Q., Robinet P., Berisha S., Smith J.D. (2021). Soat1 mediates the mouse strain effects on cholesterol loading-induced endoplasmic reticulum stress and CHOP expression in macrophages. Biochim. Biophys. Acta Mol. Cell. Biol. Lipids..

[28] Xiong W., Meng X.F., Zhang C. (2021). NLRP3 Inflammasome in Metabolic-Associated Kidney Diseases: An Update. Front. Immunol..

[29] Ritchey B., Hai Q., Han J., Barnard J., Smith J.D. (2021). Genetic variant in 3’ untranslated region of the mouse *pycard* gene regulates inflammasome activity. eLife.

[30] Tonacci A., Quattrocchi P., Gangemi S. (2019). IL33/ST2 Axis in Diabetic Kidney Disease: A Literature Review. Medicina.

[31] Liu C.L., Shen D.L., Zhu K., Tang J.N., Hai Q.M., Zhang J.Y. (2013). Levels of interleukin-33 and interleukin-6 in patients with acute coronary syndrome or stable angina. Clin. Investig. Med..

[32] Klimontov V.V., Koroleva E.A., Khapaev R.S., Korbut A.I., Lykov A.P. (2021). Carotid Artery Disease in Subjects with Type 2 Diabetes: Risk Factors and Biomarkers. J. Clin. Med..

[33] Roy S., Schweiker-Kahn O., Jafry B., Masel-Miller R., Raju R.S., O’Neill L.M.O., Correia C.R., Trivedi A., Johnson C., Pilot C. (2021). Risk Factors and Comorbidities Associated with Diabetic Kidney Disease. J. Prim. Care Community Health..

[34] Ito H., Komatsu Y., Mifune M., Antoku S., Ishida H., Takeuchi Y., Togane M. (2010). The estimated GFR, but not the stage of diabetic nephropathy graded by the urinary albumin excretion, is associated with the carotid intima-media thickness in patients with type 2 diabetes mellitus: A cross-sectional study. Cardiovasc. Diabetol..

[35] Chan W.B., Chan N.N., Lai C.W., So W.Y., Lo M.K., Lee K.F., Chow C.C., Metreweli C., Chan J.C. (2006). Vascular defect beyond the endothelium in type II diabetic patients with overt nephropathy and moderate renal insufficiency. Kidney Int..

[36] Seo D.H., Kim S.H., Song J.H., Hong S., Suh Y.J., Ahn S.H., Woo J.T., Baik S.H., Park Y., Lee K.W. (2019). KNDP StudyGroup. Presence of Carotid Plaque Is Associated with Rapid Renal Function Decline in Patients with Type 2 Diabetes Mellitus and Normal Renal Function. Diabetes Metab. J..

[37] Cardoso C.R.L., Salles G.C., Leite N.C., Salles G.F. (2019). Prognostic impact of carotid intima-media thickness and carotid plaques on the development of micro- and macrovascular complications in individuals with type 2 diabetes: The Rio de Janeiro type 2 diabetes cohort study. Cardiovasc. Diabetol..

[38] Pofi R., Giannetta E., Feola T., Galea N., Barbagallo F., Campolo F., Badagliacca R., Barbano B., Ciolina F., Defeudis G. (2022). Sex-specific effects of daily tadalafil on diabetic heart kinetics in RECOGITO, a randomized, double-blind, placebo-controlled trial. Sci. Transl. Med..

[39] Fukui M., Tanaka M., Hamaguchi M., Senmaru T., Sakabe K., Asano M., Yamazaki M., Hasegawa G., Imai S., Nakamura N. (2012). Toe-brachial index is associated more strongly with albuminuria or glomerular filtration rate than ankle-brachial index in patients with type 2 diabetes. Hypertens. Res..

[40] Lee C.C., Tsai M.C., Liu S.C., Pan C.F. (2018). Relationships between chronic comorbidities and the atherosclerosis indicators ankle-brachial index and brachial-ankle pulse wave velocity in patients with type 2 diabetes mellitus. J. Investig. Med..

[41] Draznin B., Aroda V.R., Bakris G., Benson G., Brown F.M., Freeman R., Green J., Huang E., Isaacs D., American Diabetes Association Professional Practice, Committee (2022). 11. Chronic Kidney Disease and Risk Management: Standards of Medical Care in Diabetes-2022. Diabetes Care.

[42] Bhatt D.L., Fox K.A., Hacke W., Berger P.B., Black H.R., Boden W.E., Cacoub P., Cohen E.A., Creager M.A., Easton J.D. (2006). Clopidogrel and aspirin versus aspirin alone for the prevention of atherothrombotic events. N. Engl. J. Med..

[43] Eikelboom J.W., Connolly S.J., Bosch J., Dagenais G.R., Hart R.G., Shestakovska O., Diaz R., Alings M., Lonn E.M., Anand S.S. (2017). Rivaroxaban with or without Aspirin in Stable Cardiovascular Disease. N. Engl. J. Med..

[44] Ridker P.M., Everett B.M., Thuren T., MacFadyen J.G., Chang W.H., Ballantyne C., Fonseca F., Nicolau J., Koenig W., Anker S.D. (2017). Antiinflammatory Therapy with Canakinumab for Atherosclerotic Disease. N. Engl. J. Med..

[45] Cannon C.P., Blazing M.A., Giugliano R.P., McCagg A., White J.A., Theroux P., Darius H., Lewis B.S., Ophuis T.O., Jukema J.W. (2015). Ezetimibe Added to Statin Therapy after Acute Coronary Syndromes. N. Engl. J. Med..

[46] Sabatine M.S., Giugliano R.P., Keech A.C., Honarpour N., Wiviott S.D., Murphy S.A., Kuder J.F., Wang H., Liu T., Wasserman S.M. (2017). Evolocumab and Clinical Outcomes in Patients with Cardiovascular Disease. N. Engl. J. Med..

[47] Schwartz G.G., Steg P.G., Szarek M., Bhatt D.L., Bittner V.A., Diaz R., Edelberg J.M., Goodman S.G., Hanotin C., Harrington R.A. (2018). Alirocumab and Cardiovascular Outcomes after Acute Coronary Syndrome. N. Engl. J. Med..

[48] Williams D.M., Jones H., Stephens J.W. (2022). Personalized Type 2 Diabetes Management: An Update on Recent Advances and Recommendations. Diabetes Metab. Syndr. Obes..

